# Supplementary material for: Galactomannan Pentasaccharide Produced from Copra Meal Enhances Tight Junction Integration of Epithelial Tissue through Activation of AMPK
Source: Biomedicines. 2019 Oct 14;7(4):81. doi: 10.3390/biomedicines7040081 (PMC6966651; doi:10.3390/biomedicines7040081)
Supplement: Supplementary file 1 [file biomedicines-07-00081-s001.zip › Supplementary/SupplementaryData NMR.pdf]

Supplementary data

Galactomannan pentasaccharide produced from copra meal enhances tight junction integration of epithelial tissue through activation of AMPK

Chatchai Nopvichai [1], Pawin Pongkorpsakol [2], Preedajit Wongkrasant [2], Karan Wangpaiboon [1], Thanapon Charoenwongpaiboon[1], Kazuo Ito [3], Chatchai Muanprasat [2], Rath Pichyangkura[1] *

[1] Department of Biochemistry, Faculty of Science, Chulalongkorn University, Thailand.
[2] Department of Physiology, Faculty of Science, Mahidol University, Thailand
[3] Graduate school of science, Osaka city university, Japan
* Correspondence: prath@chula.ac.th

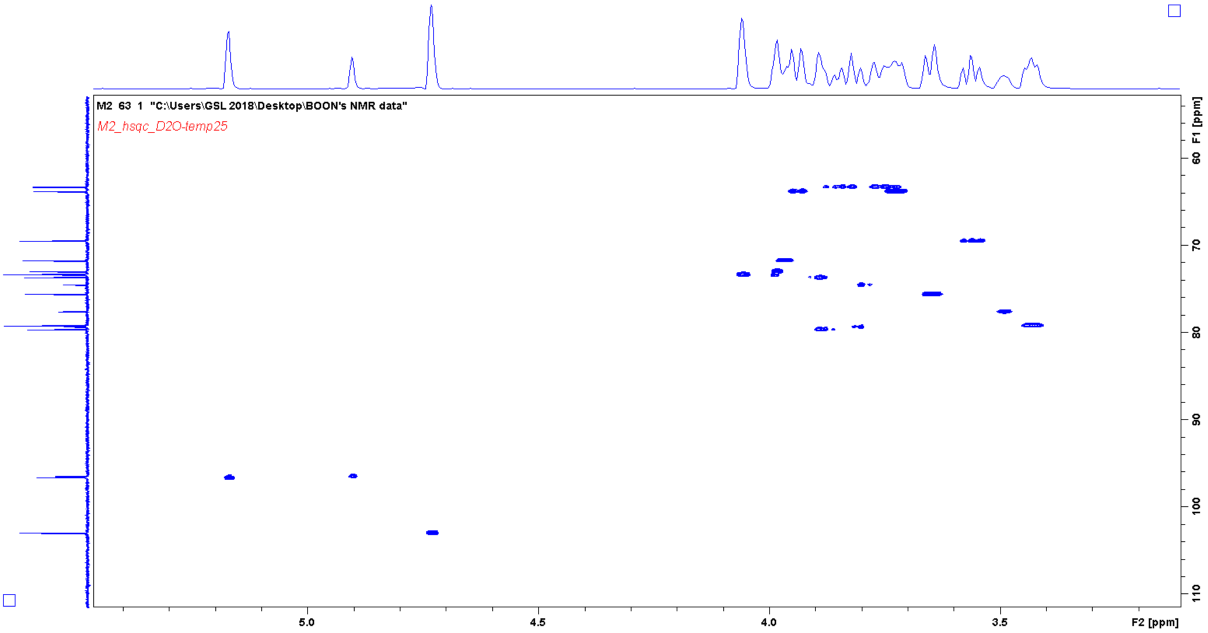

Fig.S1 HSQC spectrum of m-2.

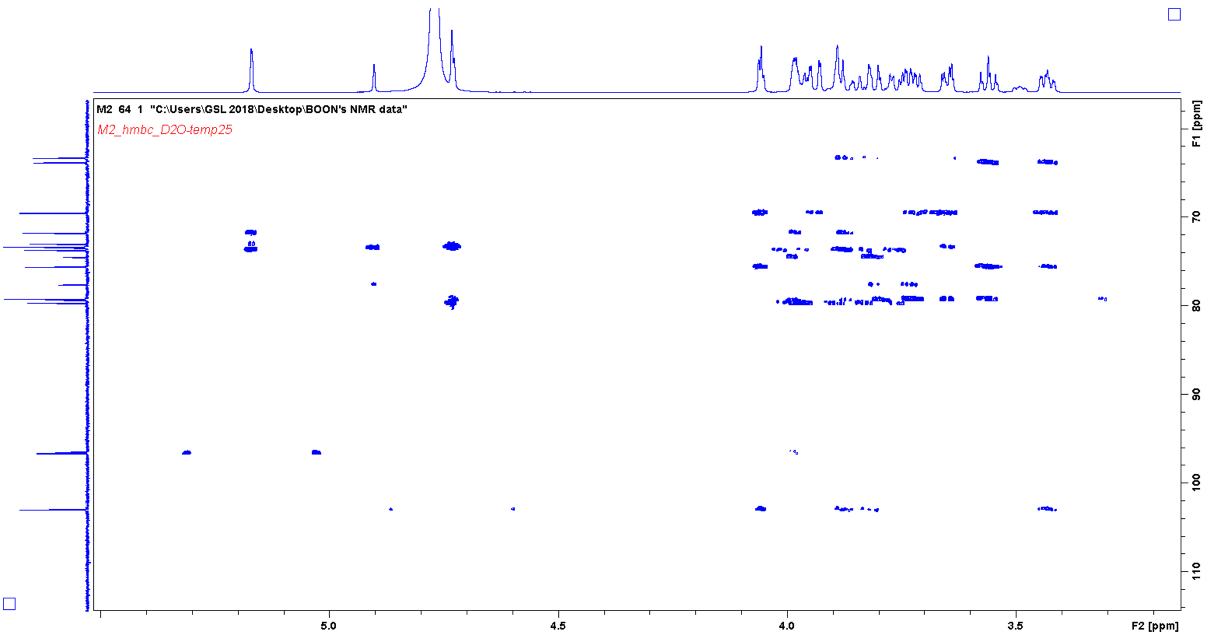

Fig.S2 HMBC spectrum of m-2.

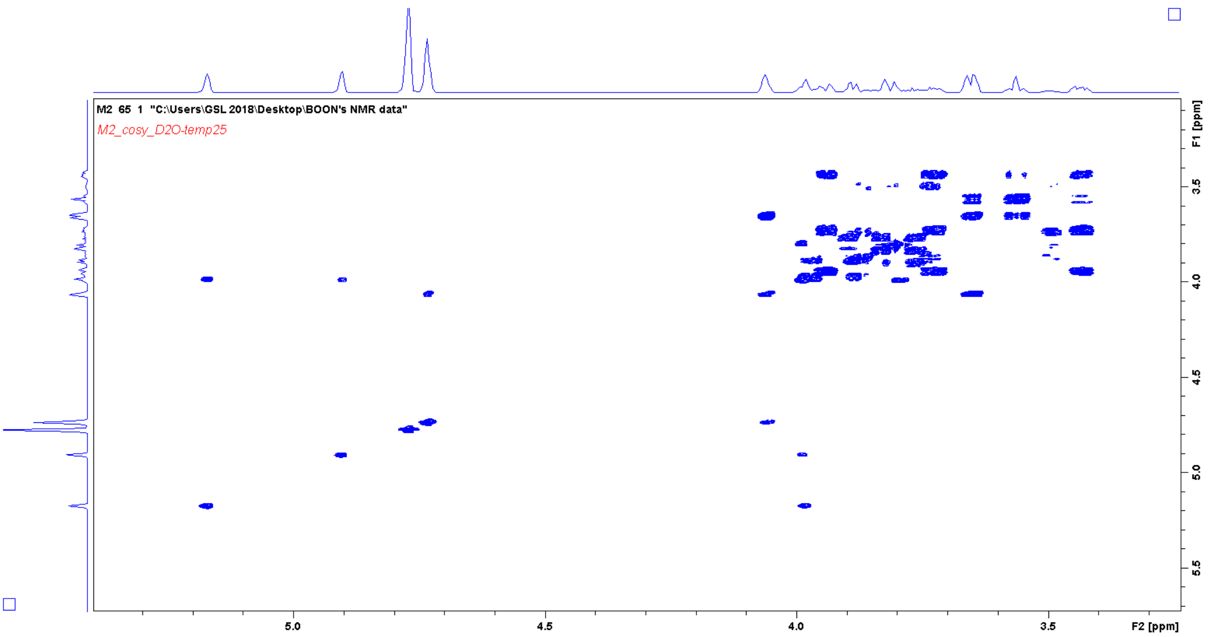

Fig.S3 COSY spectrum of m-2.

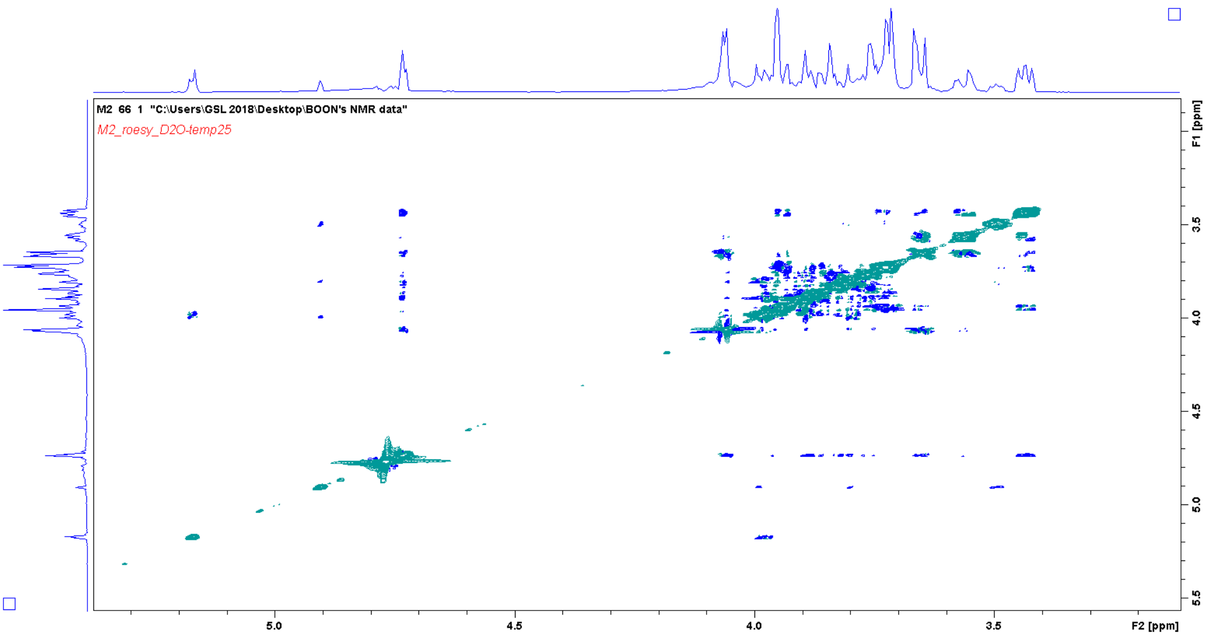

Fig.S4 ROESY spectrum of m-2.

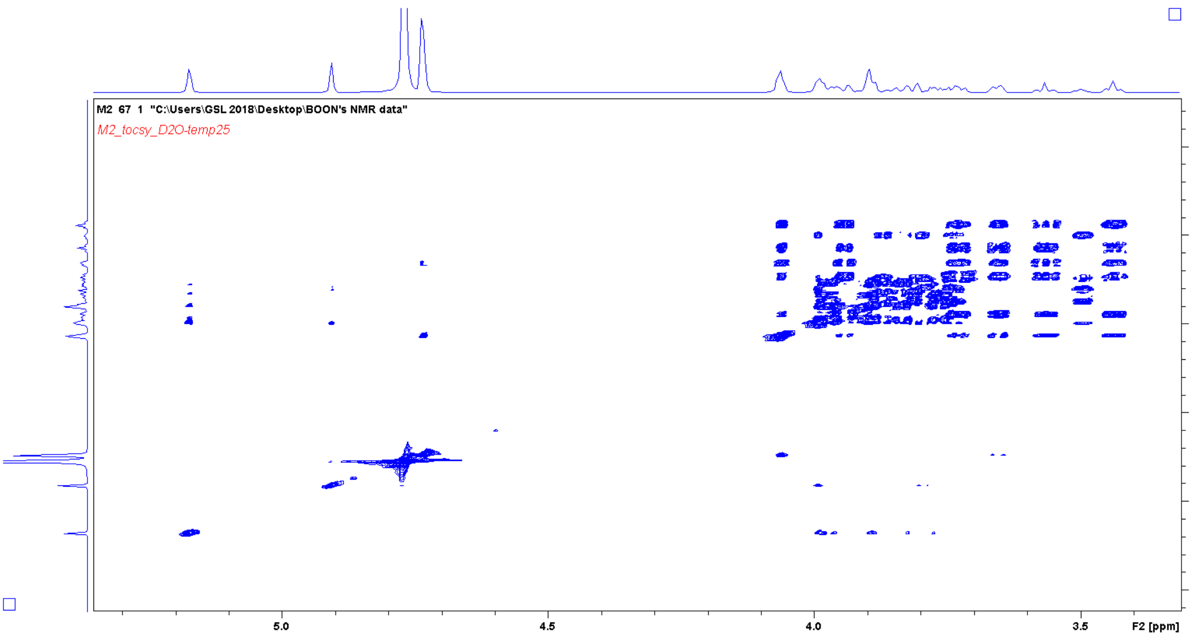

Fig.S5 TOCSY spectrum of m-2.

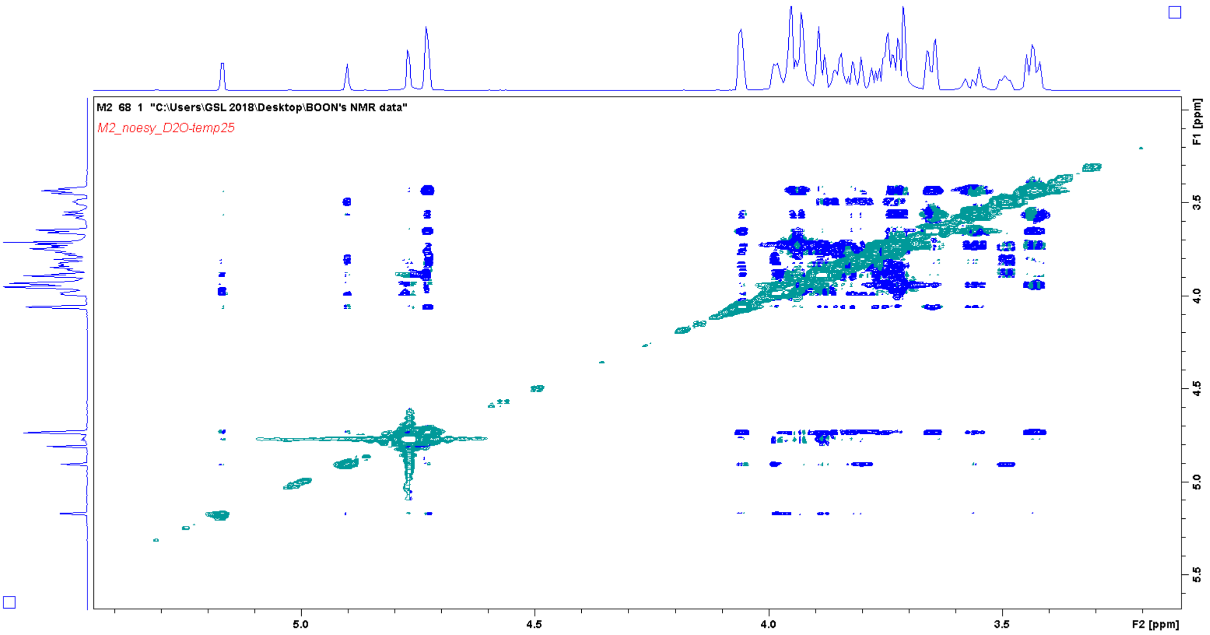

Fig.S6 NOESY spectrum of m-2.

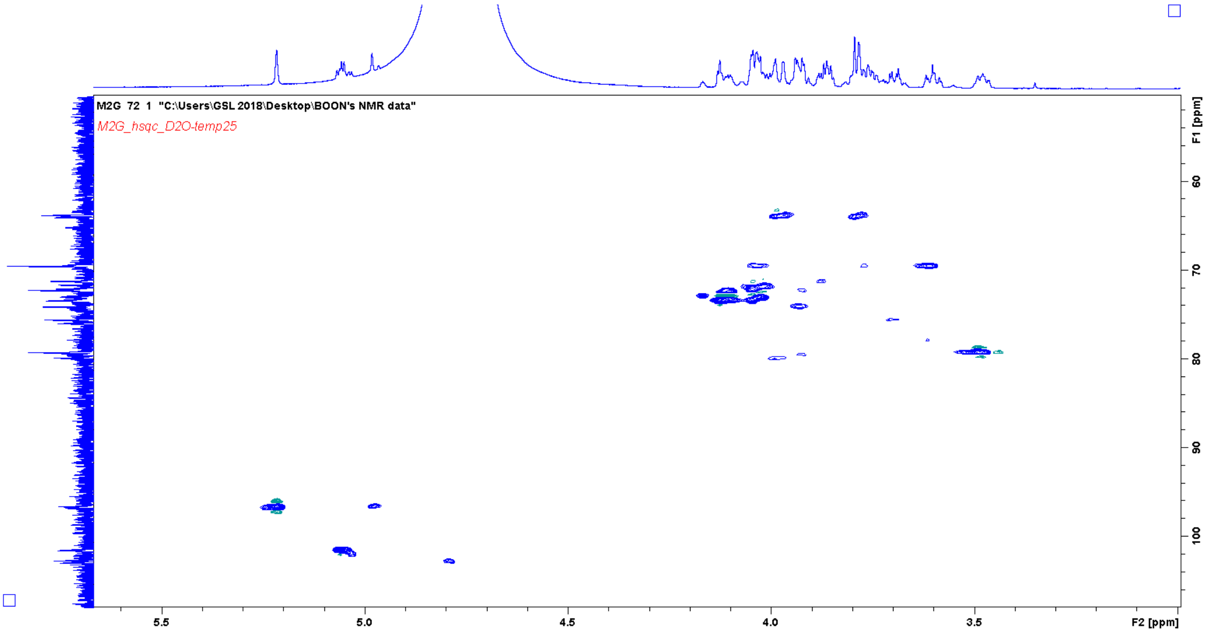

Fig.S7 HSQC spectrum of m-3.

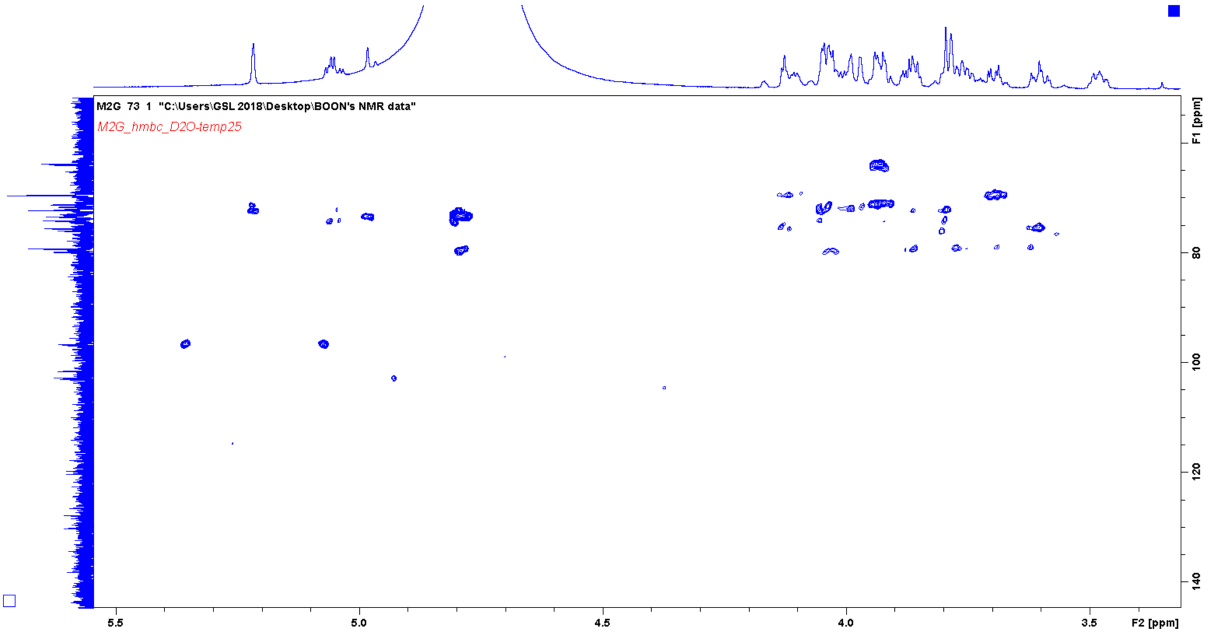

Fig.S8 HMBC spectrum of m-3.

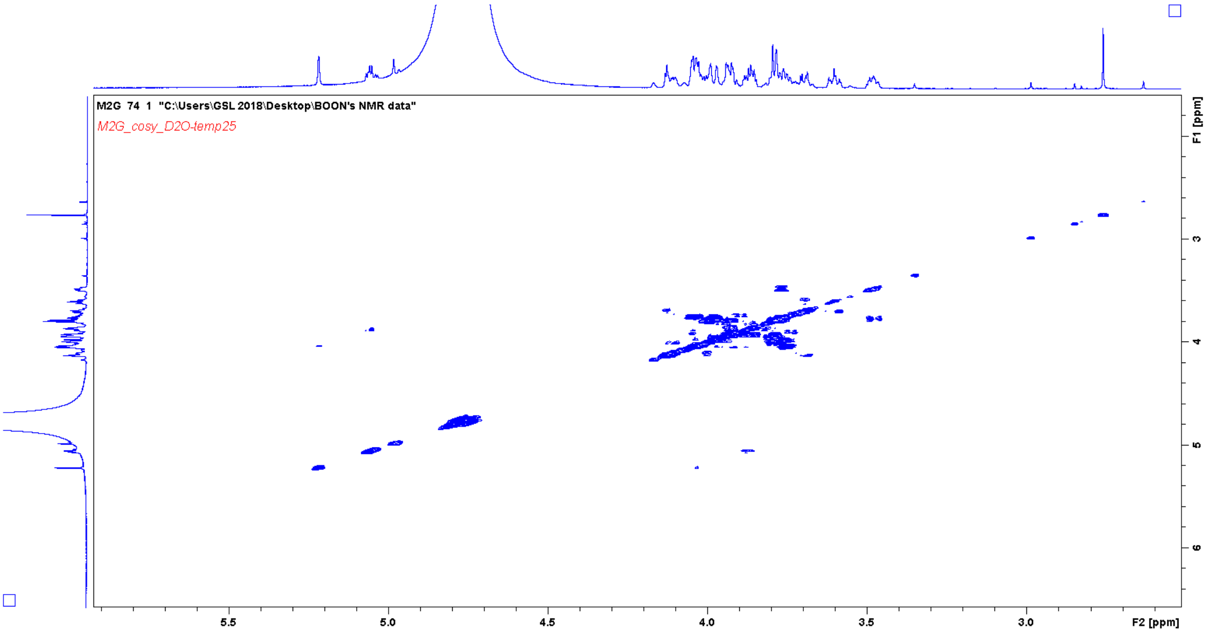

Fig.S9 COSY spectrum of m-3.

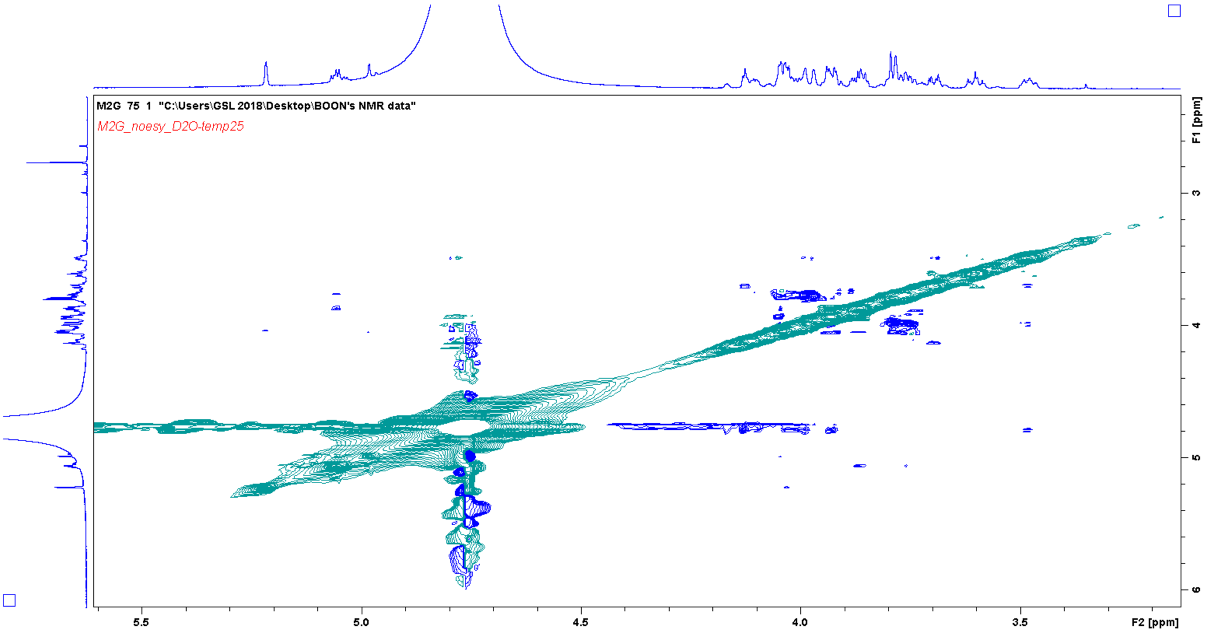

Fig.S10 NOESY spectrum of m-3.

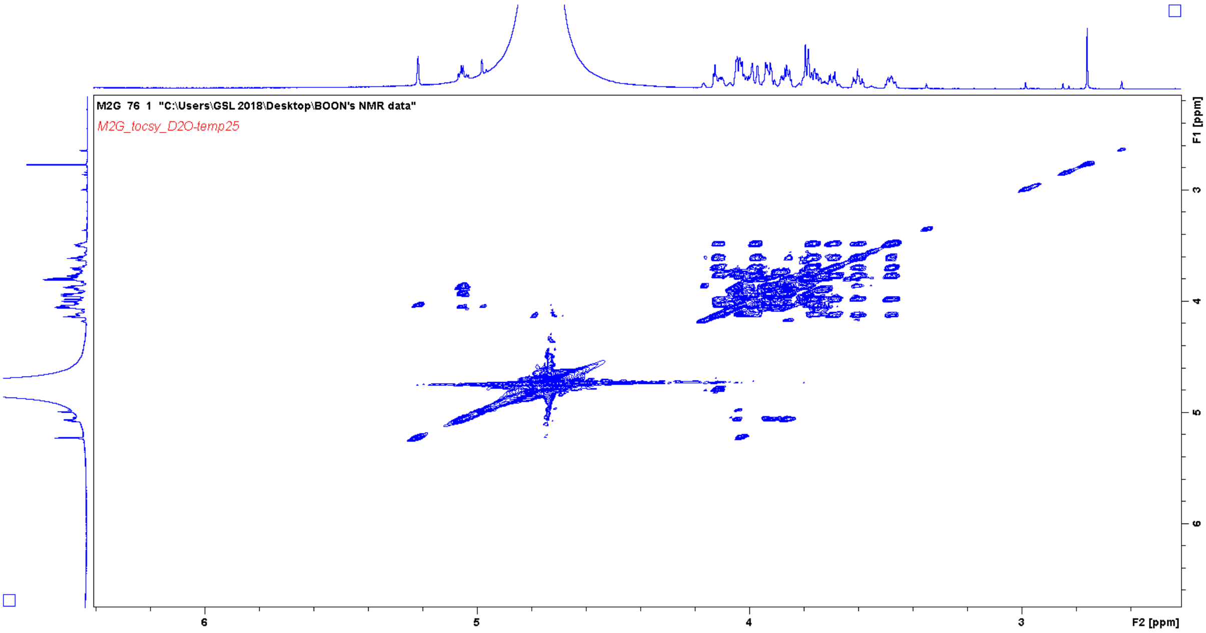

Fig.S11 TOCSY spectrum of m-3.

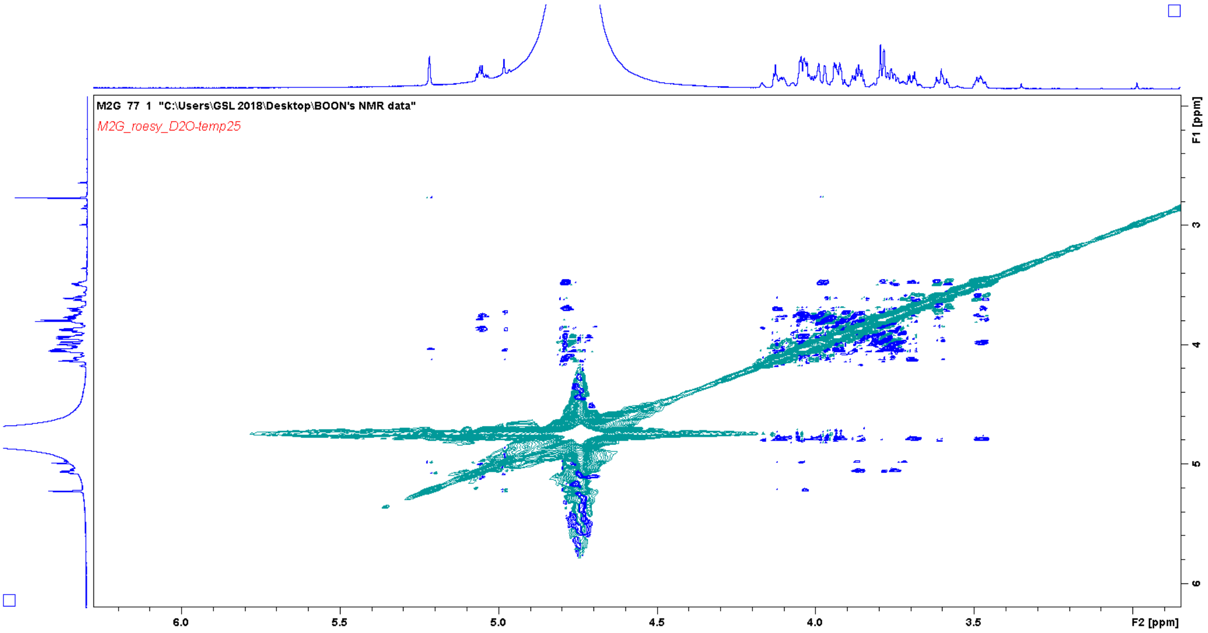

Fig.S12 ROESY spectrum of m-3.
