# Supplementary material for: Galactomannan Pentasaccharide Produced from Copra Meal Enhances Tight Junction Integration of Epithelial Tissue through Activation of AMPK
Source: Biomedicines. 2019 Oct 14;7(4):81. doi: 10.3390/biomedicines7040081 (PMC6966651; doi:10.3390/biomedicines7040081)
Supplement: Supplementary file 1 [file biomedicines-07-00081-s001.zip › Supplementary/SupplementaryData NMR.docx]

Supplementary data

Galactomannan pentasaccharide produced from copra meal enhances tight junction integration of epithelial tissue through activation of AMPK

Chatchai Nopvichai ^1^, Pawin Pongkorpsakol ^2^, Preedajit Wongkrasant ^2^, Karan Wangpaiboon ^1^, Thanapon Charoenwongpaiboon^1^, Kazuo Ito ^3^, Chatchai Muanprasat ^2^, Rath Pichyangkura^1 *^

^1^ Department of Biochemistry, Faculty of Science, Chulalongkorn University, Thailand.

^2^ Department of Physiology, Faculty of Science, Mahidol University, Thailand

^3^ Graduate school of science, Osaka city university, Japan

***** Correspondence: prath@chula.ac.th


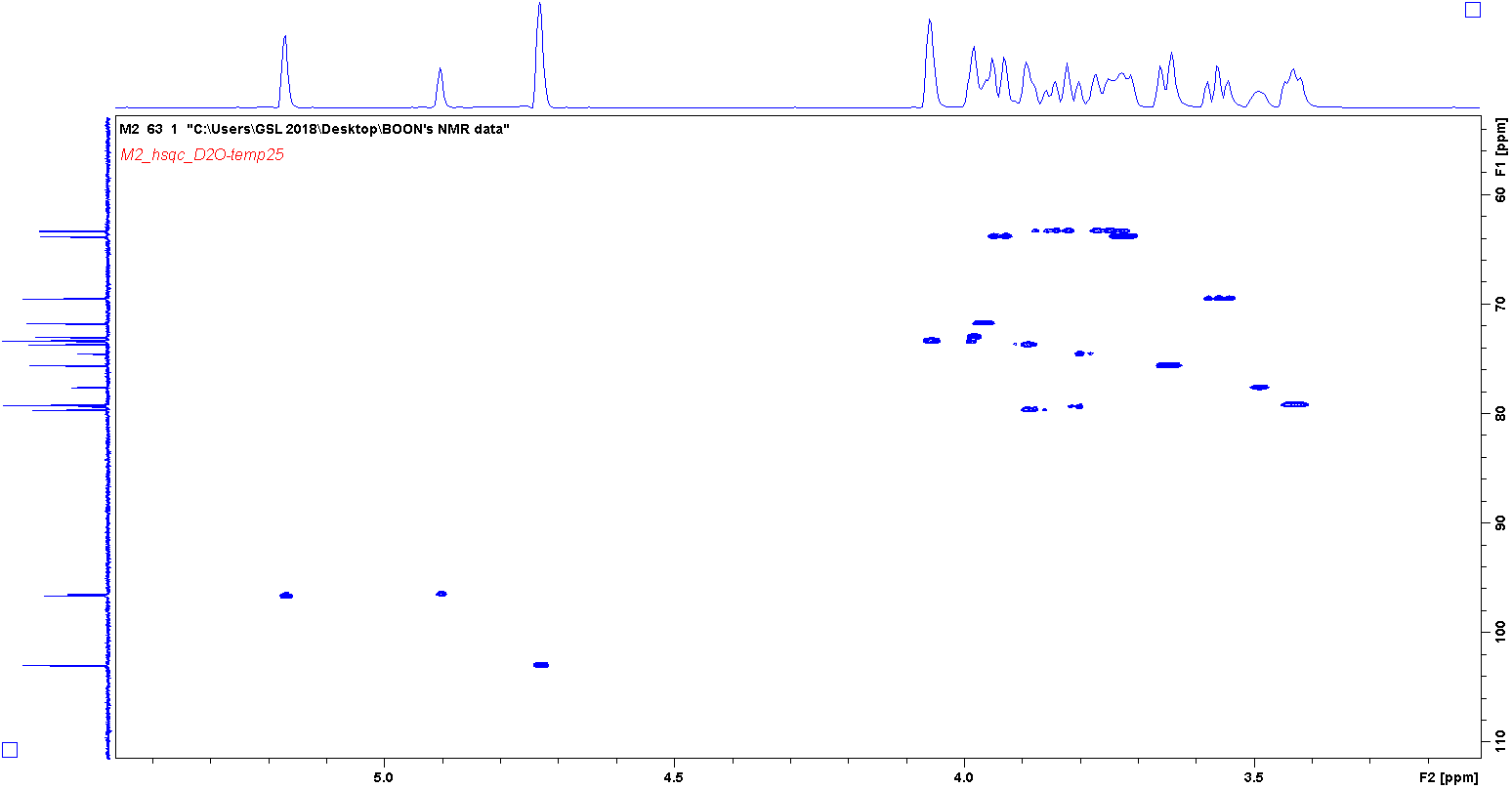


**Fig.S1 HSQC spectrum of m-2.**


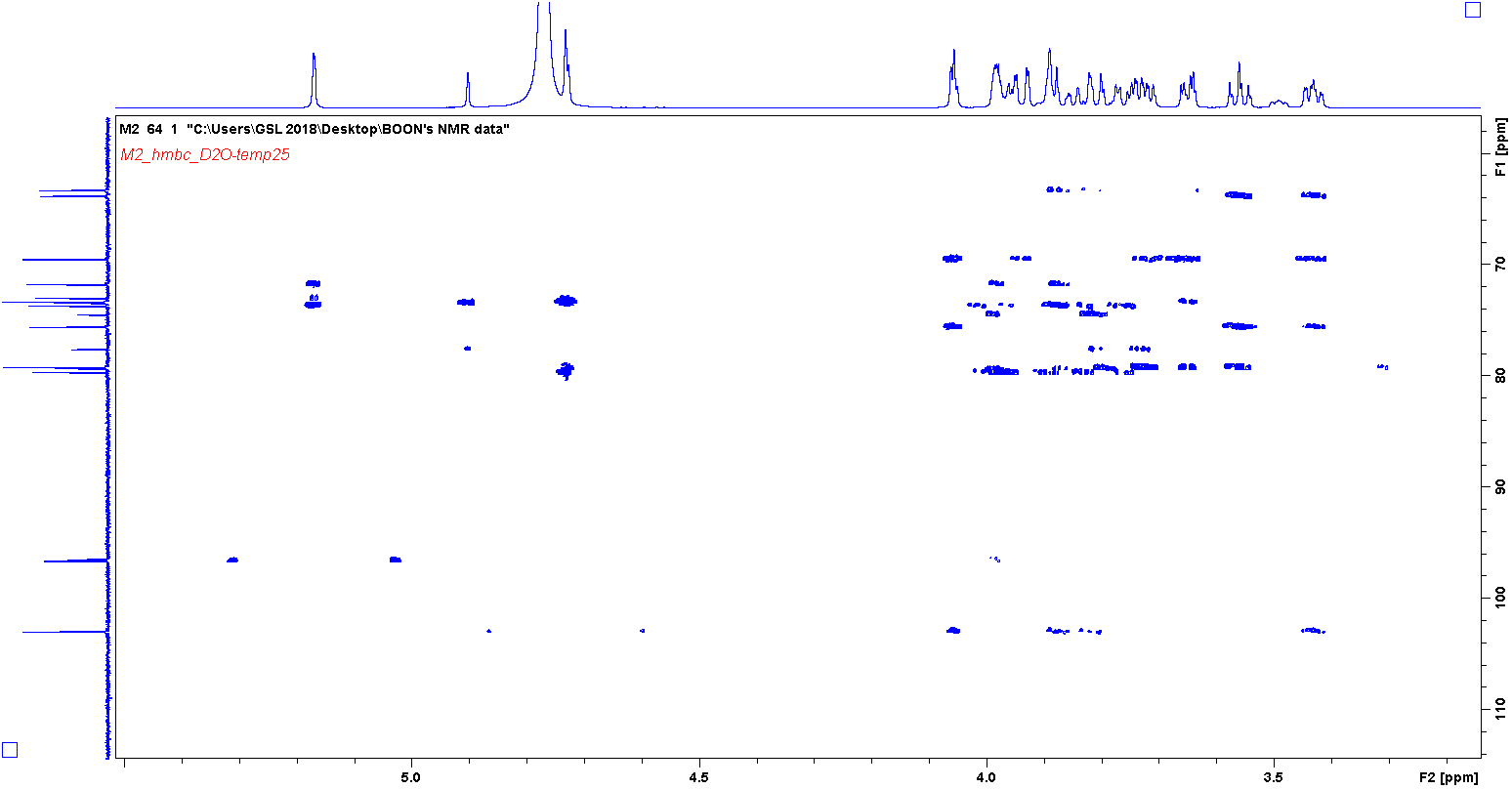


**Fig.S2 HMBC spectrum of m-2.**


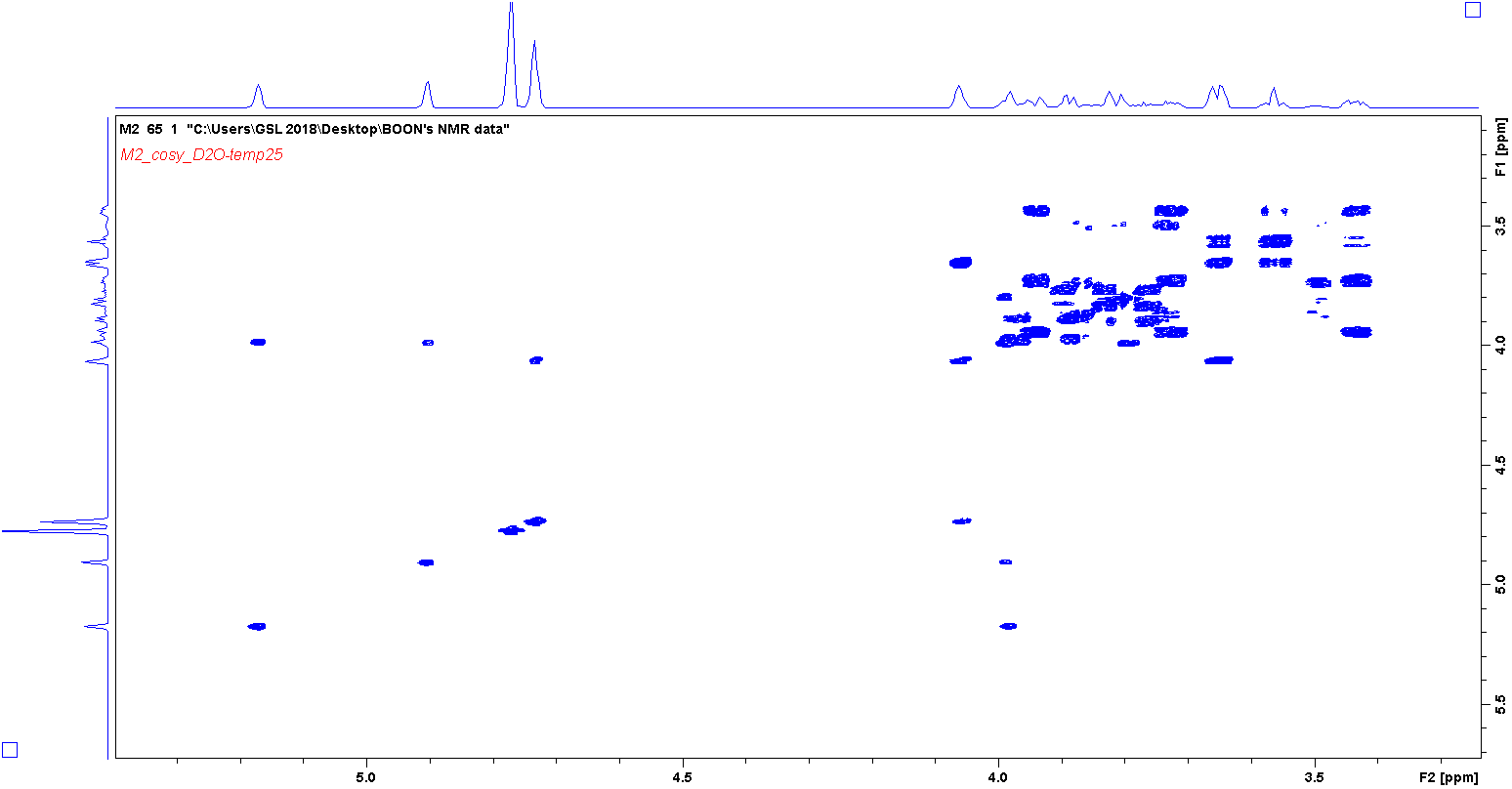


**Fig.S3 COSY spectrum of m-2.**


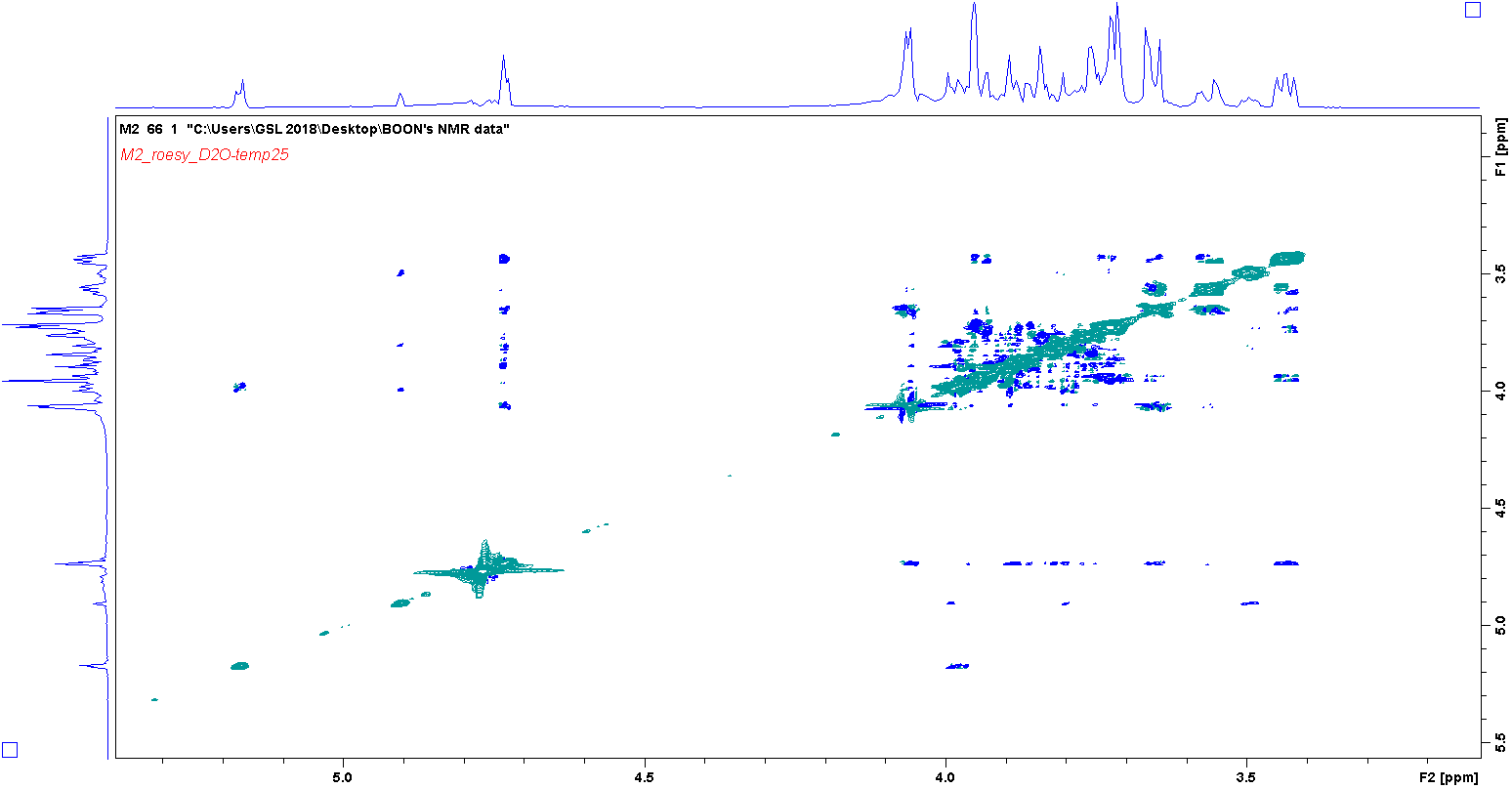


**Fig.S4 ROESY spectrum of m-2.**


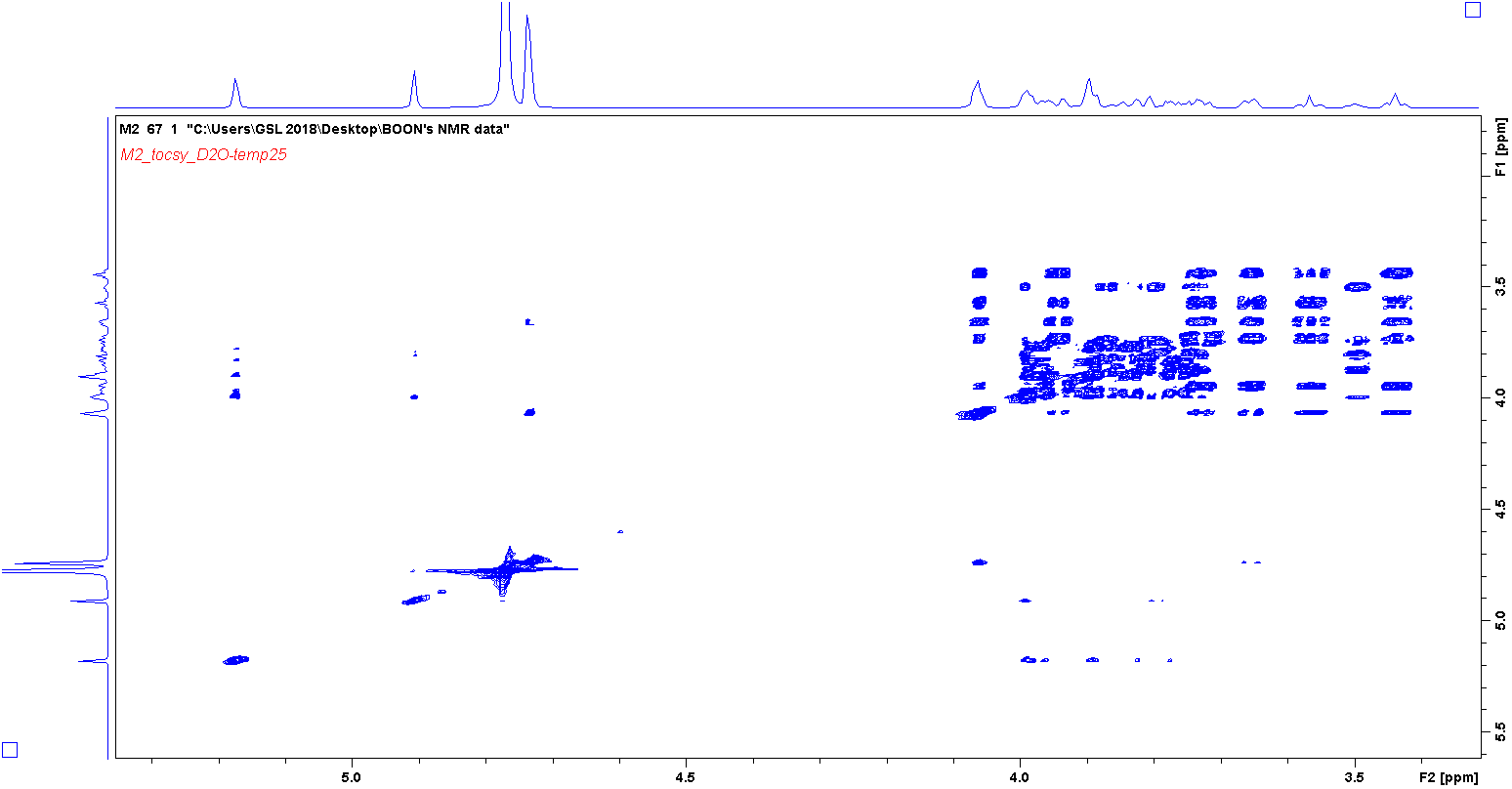


**Fig.S5 TOCSY spectrum of m-2.**


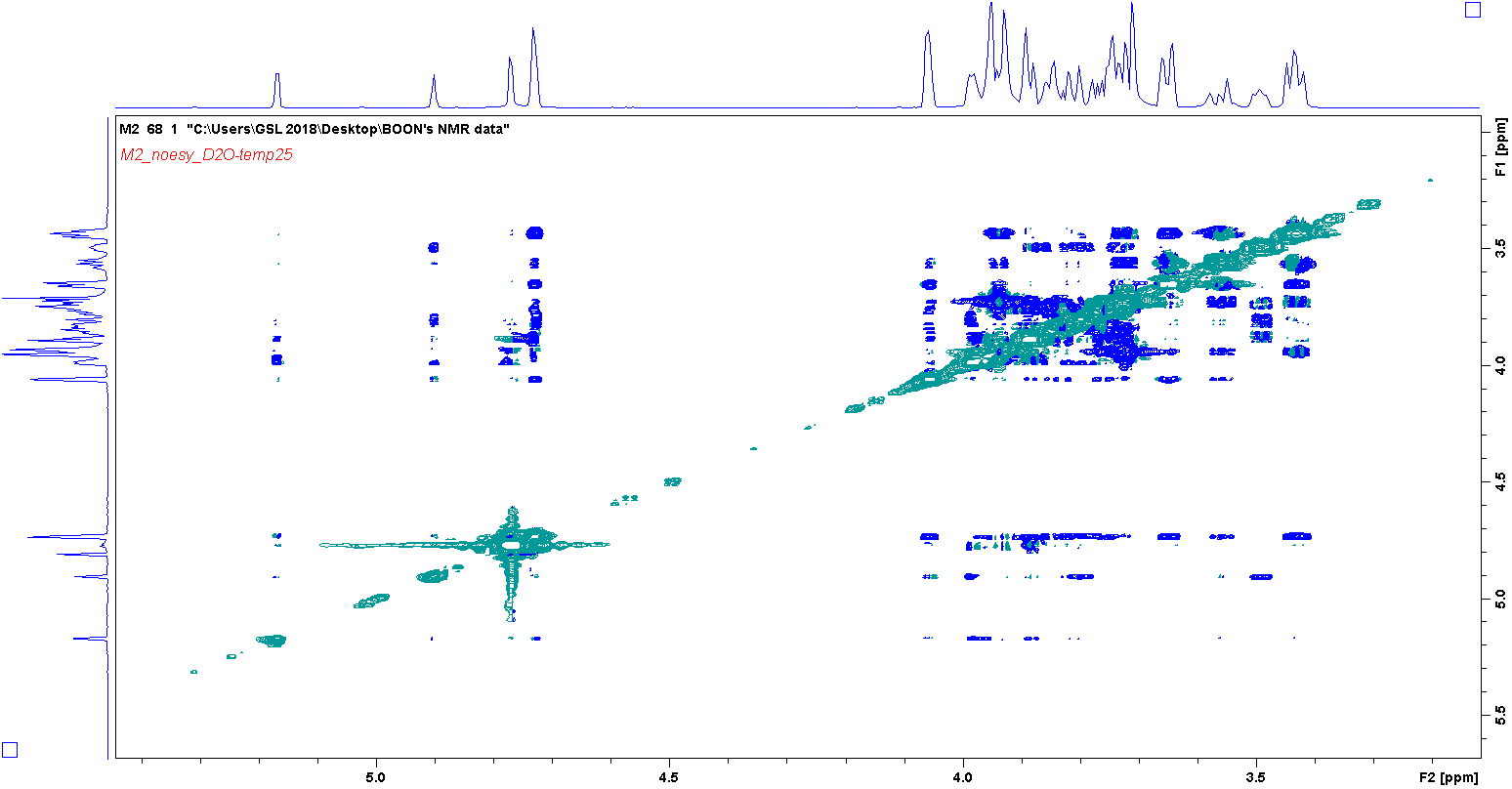


**Fig.S6 NOESY spectrum of m-2.**


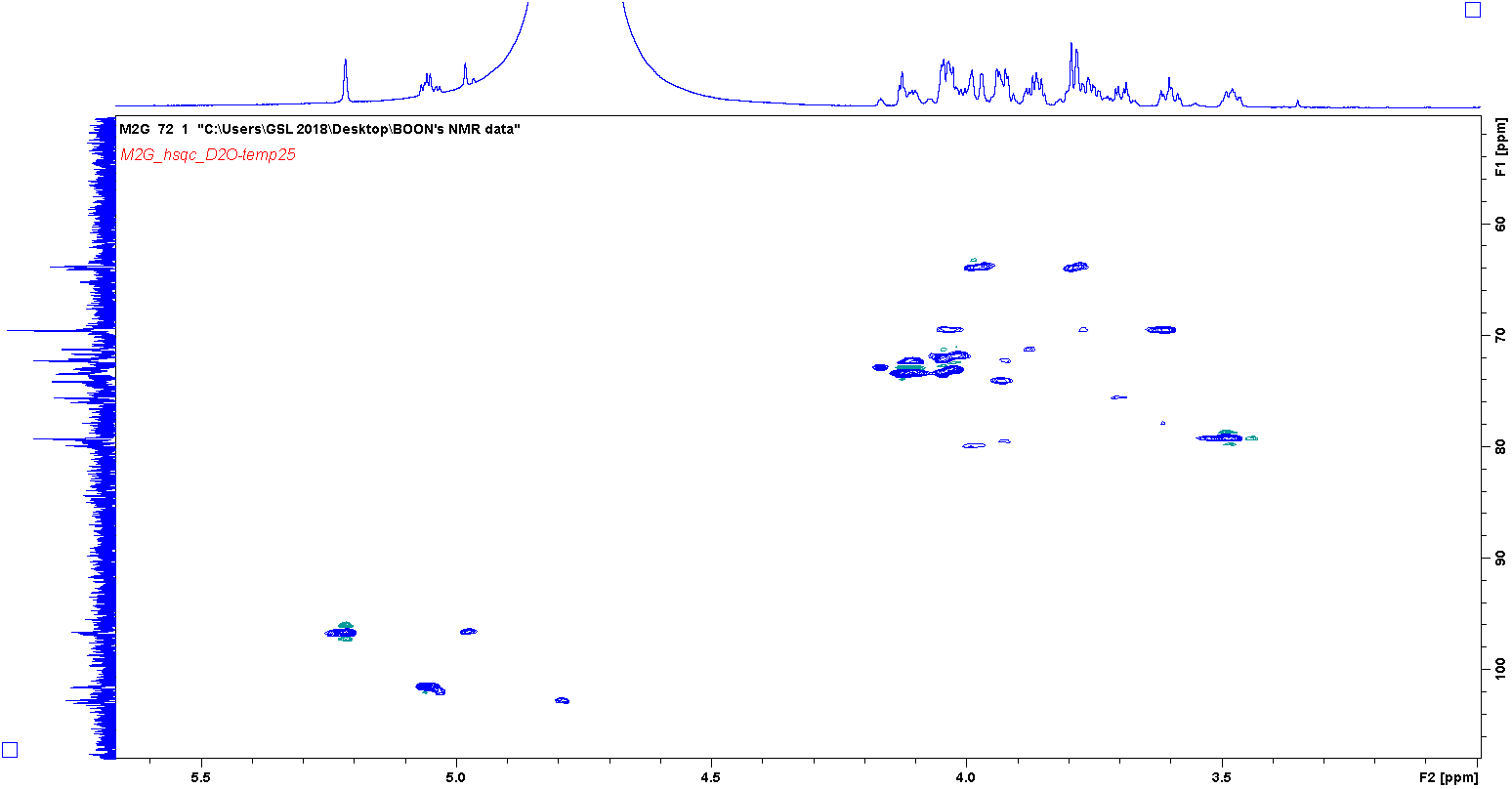


**Fig.S7 HSQC spectrum of m-3.**


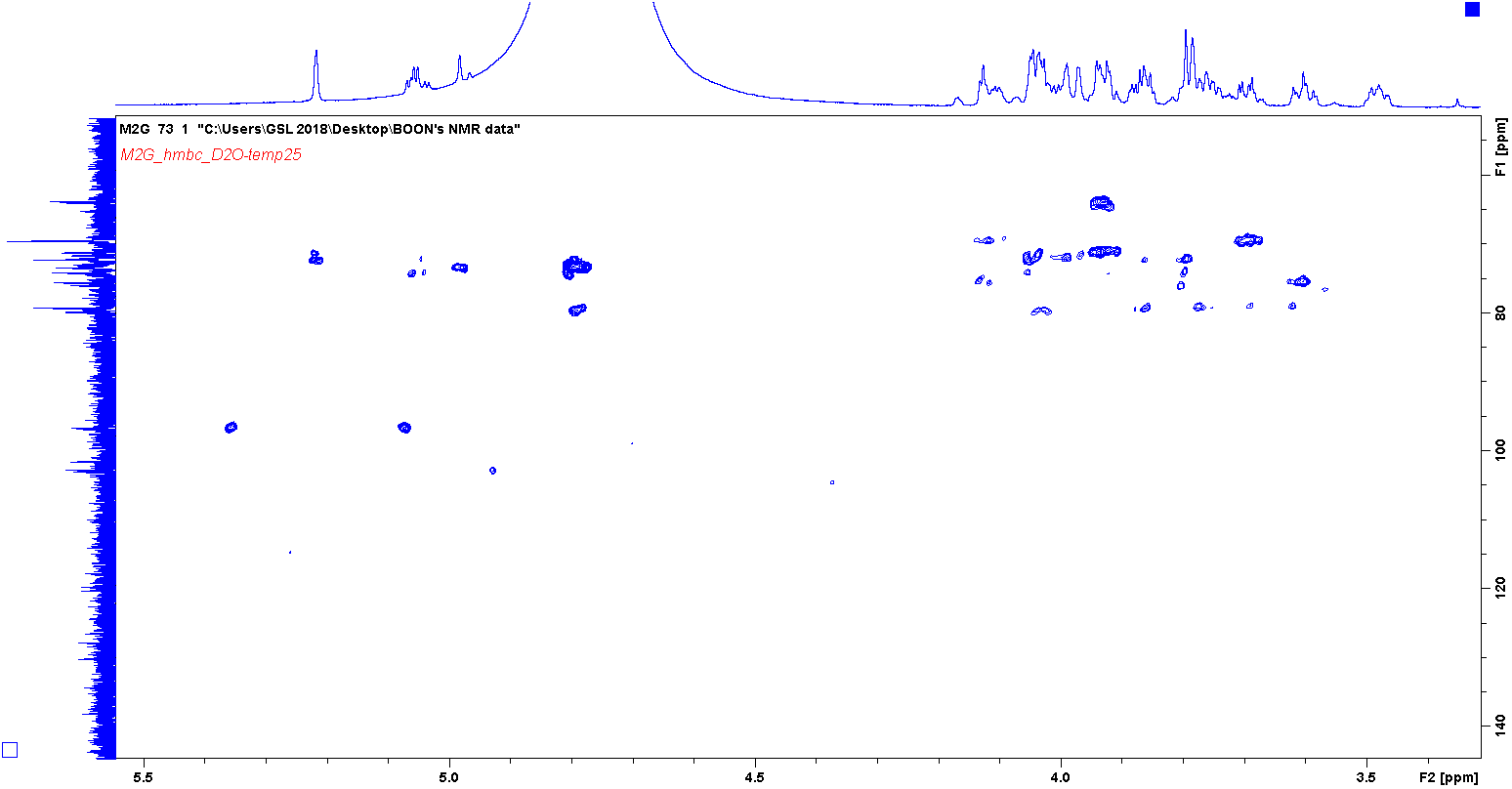


**Fig.S8 HMBC spectrum of m-3.**


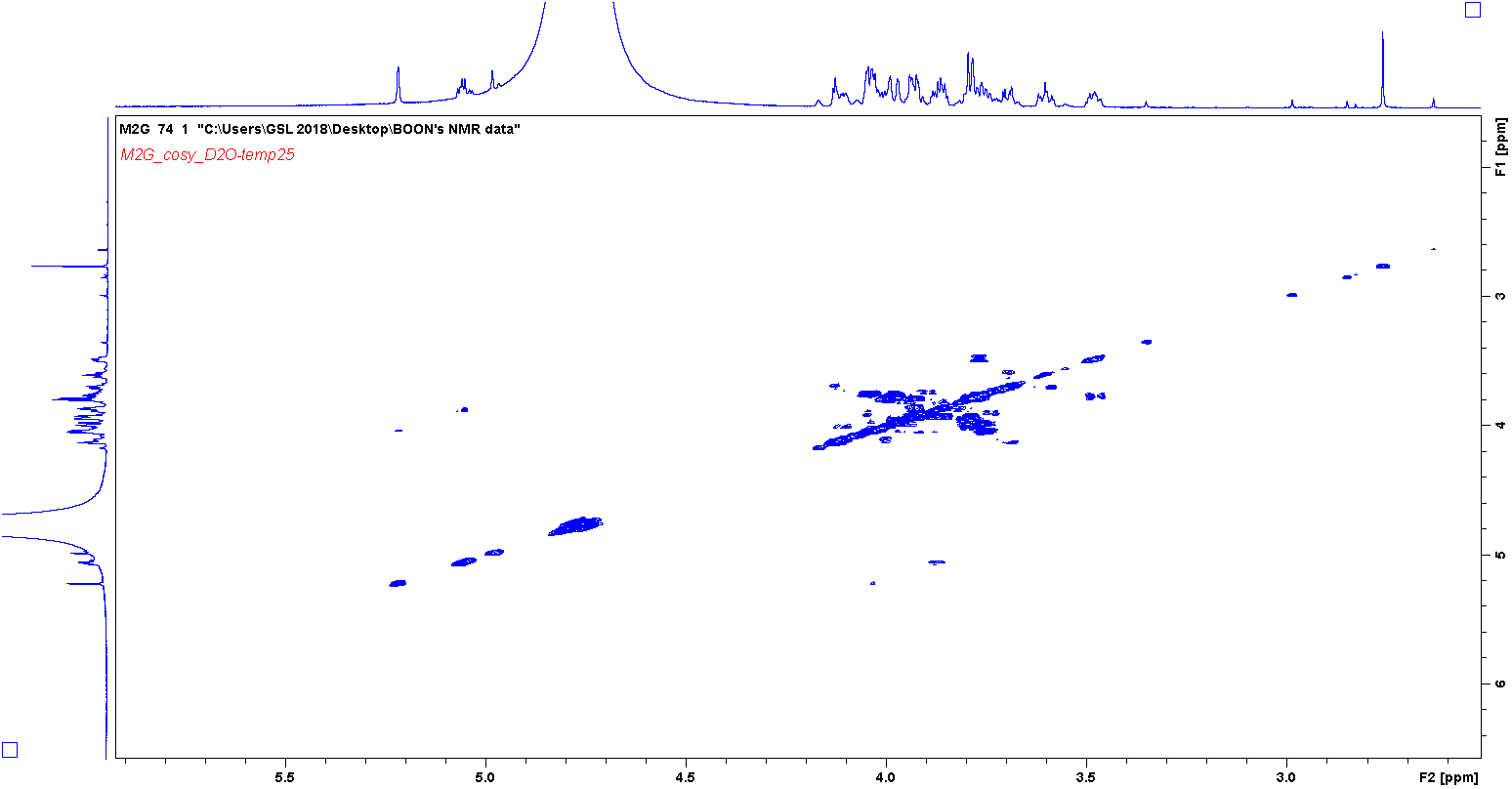


**Fig.S9 COSY spectrum of m-3.**


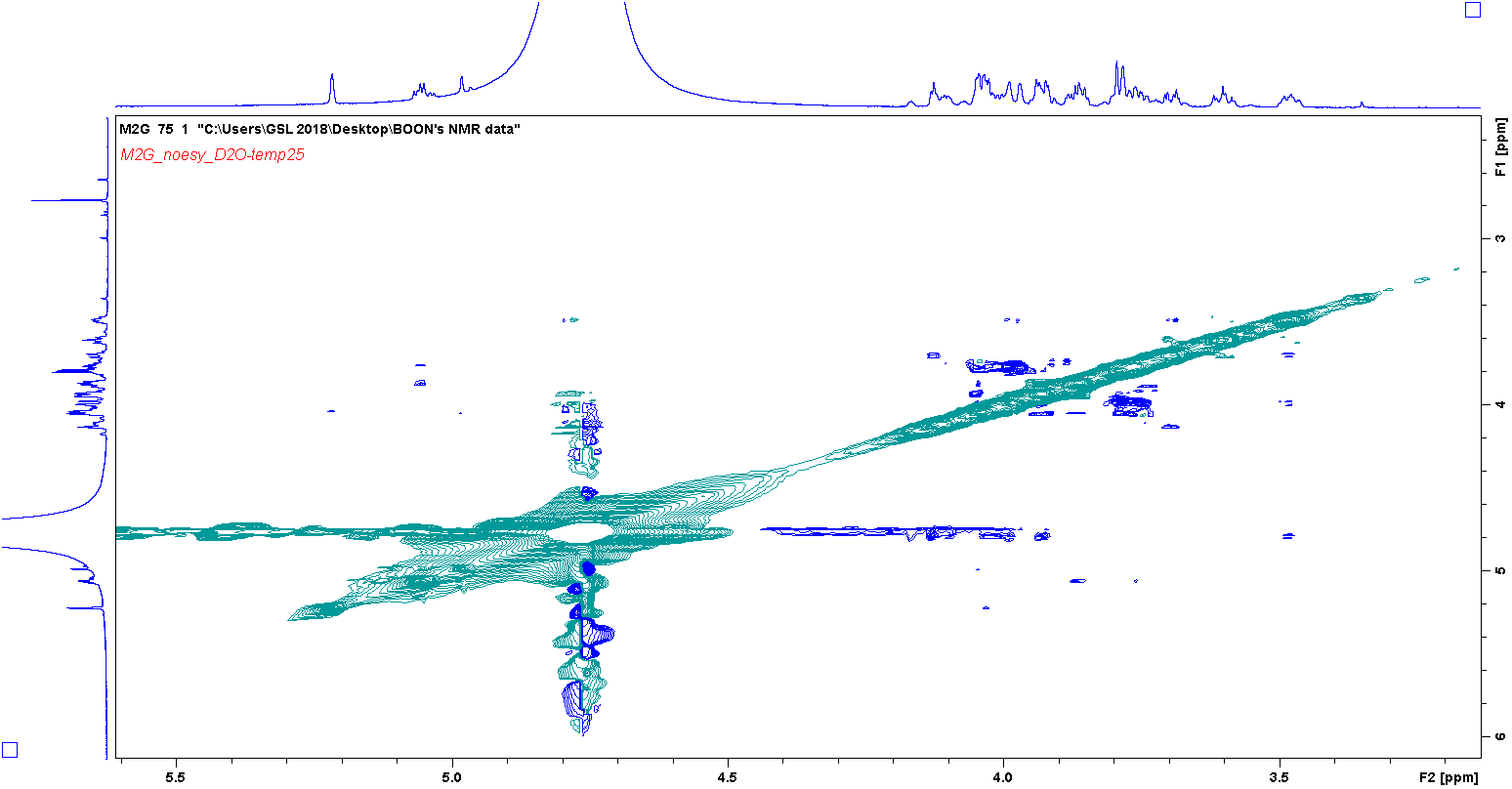


**Fig.S10 NOESY spectrum of m-3.**


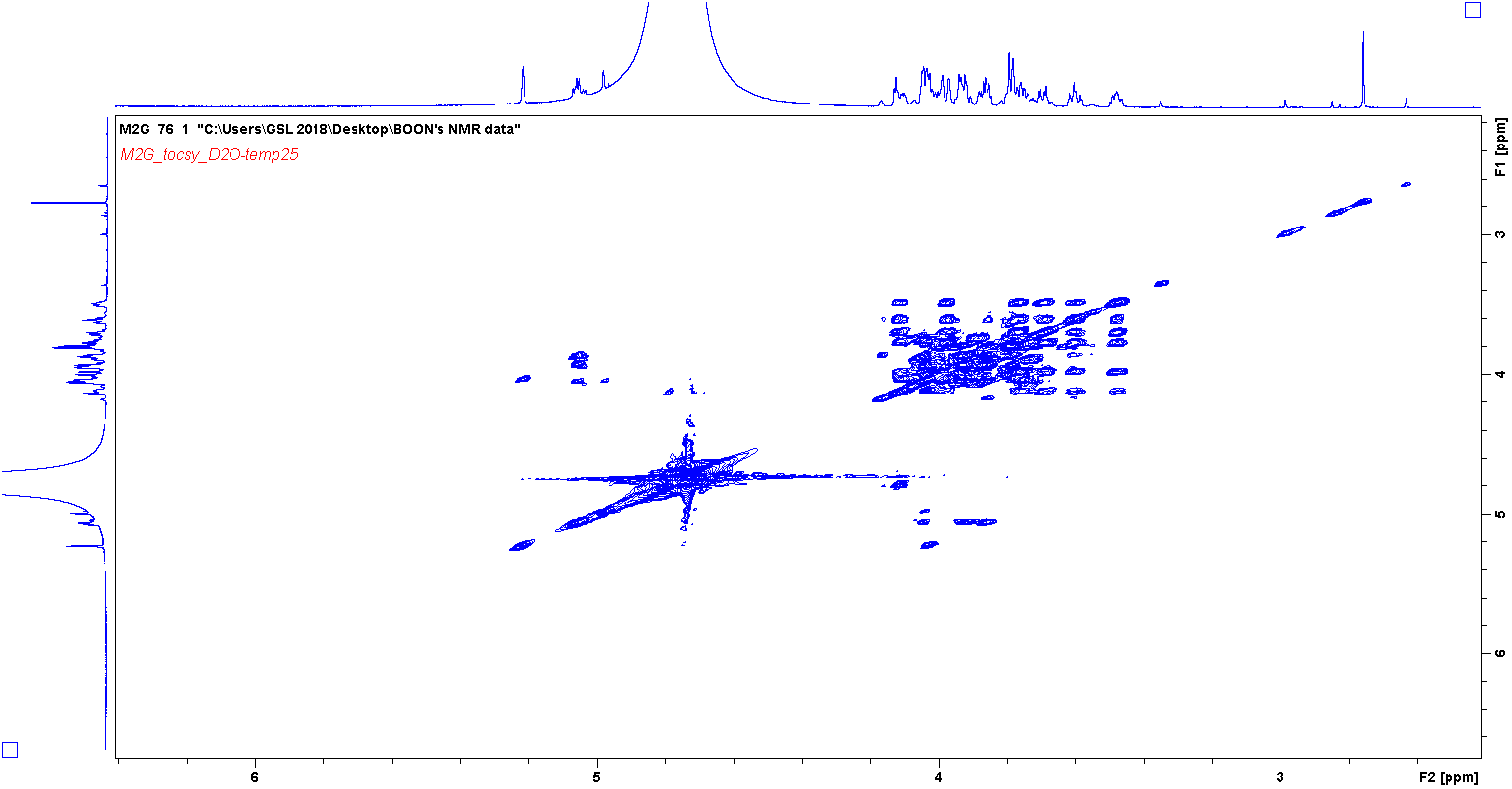


**Fig.S11 TOCSY spectrum of m-3.**


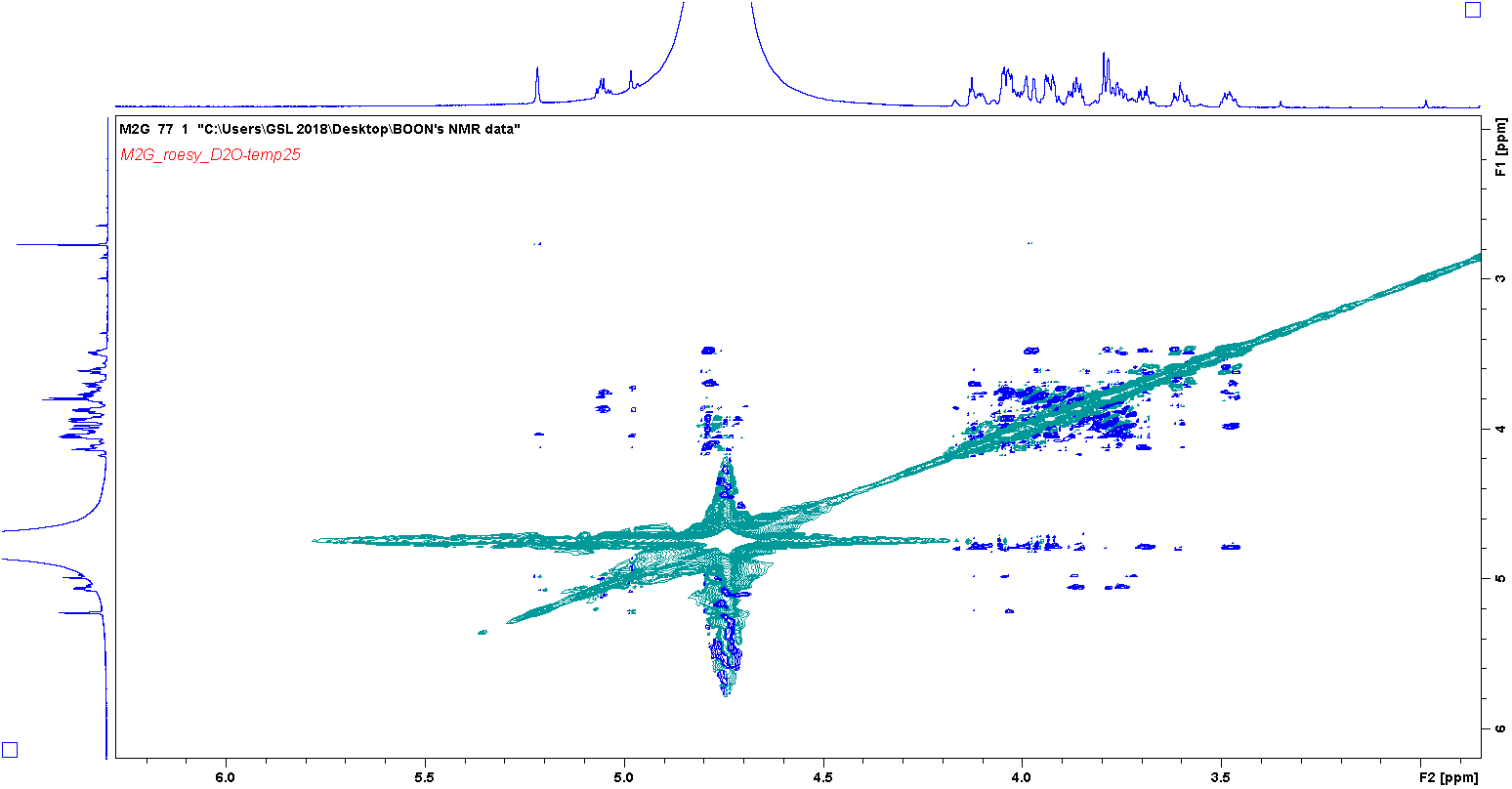


**Fig.S12 ROESY spectrum of m-3.**
